# The optimal CO_2_ concentrations for the growth of three perennial grass species

**DOI:** 10.1186/s12870-018-1243-3

**Published:** 2018-02-05

**Authors:** Yunpu Zheng, Fei Li, Lihua Hao, Arshad Ali Shedayi, Lili Guo, Chao Ma, Bingru Huang, Ming Xu

**Affiliations:** 1 School of Water Conservancy and Hydropower, Hebei University of Engineering, Handan, 056038 China; 2 Key Laboratory of Ecosystem Network Observation and Modeling, Institute of Geographic Sciences and Natural Resources Research, Chinese Academy of Sciences, 11A Datun Road, Beijing, 100101 China; 3 Department of Biological Sciences, Karakoram International University Gilgit, Gilgit, Pakistan; 4 Department of Plant Biology and Pathology, Rutgers University, New Brunswick, NJ 08901 USA; 5 Center for Remote Sensing and Spatial Analysis, Department of Ecology, Evolution and Natural Resources, Rutgers University, 14 College Farm Road, New Brunswick, NJ 08901 USA

## Abstract

**Background:**

Grasslands are one of the most representative vegetation types accounting for about 20% of the global land area and thus the response of grasslands to climate change plays a pivotal role in terrestrial carbon balance. However, many current climate change models, based on earlier results of the doubling-CO_2_ experiments, may overestimate the CO_2_ fertilization effect, and as a result underestimate the potentially effects of future climate change on global grasslands when the atmospheric CO_2_ concentration goes beyond the optimal level. Here, we examined the optimal atmospheric CO_2_ concentration effect on CO_2_ fertilization and further on the growth of three perennial grasses in growth chambers with the CO_2_ concentration at 400, 600, 800, 1000, and 1200 ppm, respectively.

**Results:**

All three perennial grasses featured an apparent optimal CO_2_ concentration for growth. Initial increases in atmospheric CO_2_ concentration substantially enhanced the plant biomass of the three perennial grasses through the CO_2_ fertilization effect, but this CO_2_ fertilization effect was dramatically compromised with further rising atmospheric CO_2_ concentration beyond the optimum. The optimal CO_2_ concentration for the growth of tall fescue was lower than those of perennial ryegrass and Kentucky bluegrass, and thus the CO_2_ fertilization effect on tall fescue disappeared earlier than the other two species. By contrast, the weaker CO_2_ fertilization effect on the growth of perennial ryegrass and Kentucky bluegrass was sustained for a longer period due to their higher optimal CO_2_ concentrations than tall fescue. The limiting effects of excessively high CO_2_ concentrations may not only associate with changes in the biochemical and photochemical processes of photosynthesis, but also attribute to the declines in stomatal conductance and nitrogen availability.

**Conclusions:**

In this study, we found apparent differences in the optimal CO_2_ concentrations for the growth of three grasses. These results suggest that the growth of different types of grasses may respond differently to future elevated CO_2_ concentrations through the CO_2_ fertilization effect, and thus potentially alter the community composition and structure of grasslands. Meanwhile, our results may also be helpful for improving current process-based ecological models to more accurately predict the structure and function of grassland ecosystems under future rising atmospheric CO_2_ concentration and climate change scenarios.

## Background

It is widely evident that global atmospheric carbon dioxide (CO_2_) concentration has dramatically increased since the nineteenth century industrial revolution, elevating by about 1.6 ppm/yr. during the past five decades [1, 2]. According to the most recent report released by the Inter-Governmental Panel on Climate Change (IPCC, 2013), global atmospheric CO_2_ levels have increased from the pre-industrial level of 280 ppm to the present level of nearly 410 ppm and the growth rate of CO_2_ concentration is projected to be accelerated with an unprecedented pace of ∼1.0 ppm/yr. [2–4]. Moreover, the global atmospheric CO_2_ concentration may even reach 1000 ppm by the end of this century and nearly 2000 ppm by the end of the next century if no effective control measures are implemented [4]. This elevated global atmospheric CO_2_ concentration may not only cause climate warming, but also cause profound impacts on the net primary productivity of agricultural and natural ecosystems [5–9].

It is well known that CO_2_ is not only one of the most important greenhouse gases, but also a critical reactant for the biochemical processes of plant photosynthesis, and thus future elevated CO_2_ concentrations may affect plant growth by altering metabolic rates [10–13]. Many studies have reported that most plants may benefit from enriched atmospheric CO_2_ concentrations through the “CO_2_ fertilization effect”. Plant growth can be boosted by absorbing more CO_2_ molecules for photosynthesis under elevated CO_2_ concentrations [10, 14–17]. For example, Wand [18] reviewed the responses of wild grasses to elevated atmospheric CO_2_ concentrations and found that elevated CO_2_ increased the total biomass of C_3_ grass species by about 50%. However, other studies have shown that the CO_2_ fertilization effect on plant growth might decline or vanish beyond certain CO_2_ concentrations [7, 19, 20], and even CO_2_ enrichment induced adverse effects on some plants when the ambient CO_2_ level was above 1000 ppm [21]. In addition, many previous studies also found that the CO_2_ fertilization effect on plants had a large variation among different species. For example, Wang [18] reported a substantial increase of the biomass of young birch tree by 59% when CO_2_ concentration was doubled from about 350 ppm to 700 ppm. By contrast, Körner et al. [22] showed that the growth and biomass of five tree species in a mature deciduous forest were barely affected by increasing CO_2_ concentration to 530 ppm based on a four-year FACE experiment. These results indicate that different plant species may have different optimal CO_2_ concentrations, and that plants with higher optimal CO_2_ concentrations are likely to benefit the most from the CO_2_ fertilization effect, and at the same time, suffer less negative impacts from future climate change, mainly due to higher nitrogen and water use efficiency [23, 24].

The CO_2_ fertilization effect on plant growth was fundamentally mediated by leaf photosynthesis [19, 25, 26], which is highly correlated with plant carbon balance [27] and biochemical composition [28, 29]. Previous studies have demonstrated that elevated CO_2_ could dramatically affect net photosynthetic rates through various processes including up-regulation or down-regulation when the growth CO_2_ below or above the optimal CO_2_ for plants. Elevated CO_2_ levels generally stimulate net photosynthetic rate through directly enhancing carboxylation rates [13, 30] while competitively reducing photorespiration and dark respiration [19, 22, 31–33]. Nevertheless, the decline of net photosynthetic rate under high CO_2_ levels may be related to changes in leaf biochemical composition associated with reductions in the amount and/or activity of Rubisco [22, 26], and increases in total non-structural carbohydrates [7, 34]. Moreover, the down-regulation of net photosynthetic rate is also associated with the availability of nutrients such as nitrogen (N), which exerts an important control over the response of plants and ecosystems in rising atmospheric CO_2_ conditions [28, 35–37]. Previous studies showed that down-regulation of photosynthesis occurred in plants grown in elevated CO_2_ and limited N indicated decreased leaf N concentration [38, 39] High N availability could alleviate the down-regulation of photosynthesis in plants under elevated CO_2_ environments [19, 26, 29].

Grasslands are an important part of terrestrial ecosystems, and account for about 20% of the earth’s land area [6, 40]. Perennial grasses are the dominant species in temperate grasslands and pastures [40], and are utilized as fine turf grass, which serves many important environmental functions including erosion control, surface water detoxification and control of allergens and diseases [41, 42]. A majority of the research investigating plant response to elevated CO_2_ have been focused on crops [43–45] or trees [26, 29, 34, 46–48] and few studies have examined the effects of elevated CO_2_ on perennial grasses [17, 19, 40]. In addition, most previous studies regarding the CO_2_ fertilization effect have focused primarily on “doubling-CO_2_ experiments” with twofold higher CO_2_ concentration of about 700 or 800 ppm than the current global CO_2_ concentration [40, 42, 45, 48]. Nevertheless, the CO_2_ fertilization effect may sustain up to about 1000 ppm for leaf photosynthesis [46, 49] and 1800 ppm for grain yield of crops [50]. For example, Xu [23] examined the optimal atmospheric CO_2_ concentration of the CO_2_ fertilization effect on the growth of winter wheat and found that the optimal atmospheric CO_2_ concentration was 894 and 968 ppm for total biomass and leaf photosynthesis. So far, few experimental studies have been conducted to examine the optimal CO_2_ concentration for maximizing the CO_2_ fertilization effect on perennial grasses, which are the most important grass species in both natural grasslands and managed turf grass. Moreover, most of the modeling projections are based on strong CO_2_ fertilization according to the conclusions from earlier “doubling-CO_2_ experiments” [29, 34]. However, it should be noted that in the future, continuously rising atmospheric CO_2_ concentrations may substantially lower the CO_2_ fertilization effect when the atmospheric CO_2_ concentration rises beyond the optimal CO_2_ level [23]. As a result, many current climate change models based on earlier results of the doubling-CO_2_ experiments may overestimate the CO_2_ fertilization effect and underestimate the potential risks that climate change poses on global grasslands when the atmospheric CO_2_ concentration goes beyond the optimal CO_2_ level. Therefore, identifying optimal CO_2_ concentrations and understanding the mechanisms that determine these optima are not only critical to accurately estimating the impacts of climate change on global grassland production, but also have important significance for policy implementations under future climate change scenarios. Therefore, this study was conducted based on the following objectives: (1) investigate the effects of elevated CO_2_ concentrations on the growth of three perennial grass species, (2) examine the optimal CO_2_ concentration for maximizing the CO_2_ fertilization effect of these grasses, and (3) explore potential mechanisms that determine the optimal CO_2_ concentrations for the growth of perennial grasses.

## Methods

### Plant materials and growing conditions

Three grass species, tall fescue (*Festuca arundinacea* Schreb.), perennial ryegrass (*Lolium perenne* L.), and Kentucky bluegrass (*Poa pratensis* L.), were collected using a golf-hole cutter (10 cm diameter × 20 cm long) to ensure the same aboveground and belowground biomass of each species from field plots in the research farm at Rutgers University (Adelphia, NJ, USA). These grasses were irrigated with groundwater once a week in the field research farm to maintain a 10-cm soil surface moisture of about 40% (% volume) during the growing season. Then the collected plants were transplanted into pots (10 cm diameter × 40 cm long) filled with fritted clay and maintained in a greenhouse with an average temperature of 21/16 °C (day/night) and about 800 μmol photon m^− 2^ s^− 1^ Photosynthetic Active Radiation (PAR) in natural sun light, and 65% relative humidity for 70 d (May–June 2012) to establish canopy and root system. During the establishment period, grasses were irrigated daily to water-holding capacity and fertilized twice per week with half-strength Hoagland’s solution [51]. We trimmed grasses once a week to maintain a canopy height of 5 cm during the canopy development and root establishment period. Then the plants were trimmed to a 2-cm canopy height and moved to growth chambers (Environmental Growth Chamber) with temperatures set at 21/18 °C (day/night), 60–70% Relative Humidity (RH), light level at grass canopy of 1000 μmol m^− 2^ s^− 1^ PAR, and a 12-h photoperiod for 2 weeks prior to the CO_2_ treatment. During the eight weeks of the CO_2_ treatment, these grasses were maintained under the same environmental factors as before the start of CO_2_ treatment, such as chamber temperature of 21/18 °C (day/night), relative humidity of 60–70%, light level at the grass canopy of 1000 μmol m^− 2^ s^− 1^ PAR, and 12-h photoperiod (6:00–18:00). In addition, the grasses were also well-watered with daily irrigation and fertilized with half-strength Hoagland’s solution twice a week.

### Treatments and experimental design

We exposed grasses to five CO_2_ treatments: ambient concentration (400 ± 10 ppm) or elevated concentrations (600, 800, 1000, and 1200 ± 10 ppm). In order to minimize confounding effects of environmental variation between different chambers, we randomly changed the CO_2_ concentration of each growth chamber every three days, and then relocated the CO_2_ treated grasses to the growth chambers with corresponding CO_2_ concentrations. The experiment was arranged in a randomized complete block design with four replicates (pots) per treatment. The ambient and elevated CO_2_ concentrations within the chambers were maintained through an automatic CO_2_ control system connected to a CO_2_ source-tank containing 100% research-grade CO_2_ (Airgas, Inc.). The CO_2_ concentrations inside the chambers were continuously monitored through an infrared gas analyzer (LI-820; LICOR, Inc., Lincoln, NB, USA) connected to a computer logger maintaining the CO_2_ concentration within 10 ppm of the ambient and elevated target levels.

### Plant biomass measurements

We trimmed the plants to a 2-cm canopy height again at 14, 28, 42, and 56 days after the CO_2_ treatments. The trimmed leaves were collected and oven dried at 80 °C for 7 days, and the dry weights were subsequently measured. The dry weights of leaves collected at 14, 28, 42, and 56 days of CO_2_ treatment were put together for calculating shoot biomass during the CO_2_ treatment period. At the end of the treatment period (56 days), all plant samples were destructively removed for an analysis of root biomass accumulation. The roots were severed from the shoots at the soil line and washed to make free of fritted clay medium. All of the washed roots were then oven dried at 80 °C for 3 days, and the dry weights were subsequently measured.

### Leaf gas exchange measurements

Leaf gas exchange measurements were performed at the end of the CO_2_ treatment period (56 days). Five fully expanded leaves were randomly selected and arranged in a 2 × 3 cm^2^ cuvette chamber attached to a portable photosynthetic system (LI-6400; LICOR, Inc.). Before each measurement, leaves were equilibrated in the cuvette at saturating PPFD (1000 μmol photon m^− 2^ s^− 1^), the growth CO_2_ level, the target temperature and Vapor Pressure Deficit (VPD). CO_2_ concentrations in the cuvette were controlled using an injector system (LI-6400, LI-COR Inc.), which utilizes a CO_2_ mixer and compressed CO_2_ cartridges sealed with plasticene to prevent leakage. Then, the photosynthesis vs intercellular CO_2_ (*A*_n_-*C*_i_) curves were measured at cuvette chamber CO_2_ of 50, 100, 150, 200, 300, 400, 600, 800, 1000, 1200, and 1400 ppm. Data from *A*_n_-*C*_i_ curves were used to compare treatment effects on the light-saturated net photosynthetic rates at ambient or elevated CO_2_ (*A*_n_), the maximum carboxylation rate of Rubisco (*V*_cmax_), and the maximum capacity of electron transport mediated ribulose bisphosphate (RuBP) regeneration (*J*_max_). An estimation method was used to obtain *V*_cmax_ and *J*_max_ for each observed *A*_n_-*C*_i_ curve [52]. Meanwhile, stomatal conductance (*g*_s_), and transpiration rate (*T*_r_) were also determined with the portable photosynthesis system (LI-6400; LICOR, Inc.). Water Use Efficiency (WUE) was determined by the values of the net photosynthetic rate (*A*_n_) and transpiration rate (*T*_r_) according to the formula WUE = *A*_n_ / *T*_r_.

### Biochemical analysis

After the CO_2_ treatment period (56 days), the leaves and roots for analyzing Total Non-structural Carbohydrates (TNC) were sampled at midday, immediately frozen in liquid nitrogen and stored at − 80 °C until freeze-drying. Freeze-dried tissues were then ground to fine powder with a ball mill (MM2, Fa. Retsch, Haan, Germany), applied desiccant and stored at 20 °C. Total carbon (C) and nitrogen (N) contents in leaves and roots were determined using an elemental analyzer (Vario Max CN, Elemnetar Corp., Germany). Glucose, fructose, sucrose and starch concentrations were determined spectrophometrically (UV-1750, Shimadzu Corp., Tokyo, Japan), using a glucose kit (GAHK-20, Sigma, St Louis, MO, USA). Phospho-glucose isomerase (P5381–1 KU, Sigma) and invertase (I-4504, Sigma) were used to convert fructose to glucose and sucrose to glucose respectively. Biochemical analyses were repeated five times and expressed on a percentage dry matter basis for each.

### Data analysis

The raw data from the leaf photosynthesis measurements was cleaned and processed in Excel spreadsheets where the non-linear *A*_n_-*C*_i_ curve fitting was performed as in Sharkey et al. (2007) [52]. The net assimilation rate (*A*_n_) versus intercellular CO_2_ concentration (*A*_n_-*C*_i_ curve), were fitted to estimate the maximum carboxylation rate (*V*_cmax_), maximum electron transport rate (*J*_max_) based on the measurements of *A*_n_-*C*_i_ curves. In addition, linear and non-linear (quadratic equations) regressions were employed to examine relationships between CO_2_ concentration and other variables.

## Results

### Elevated CO_2_ effects on plant biomass

**Fig. 1:**
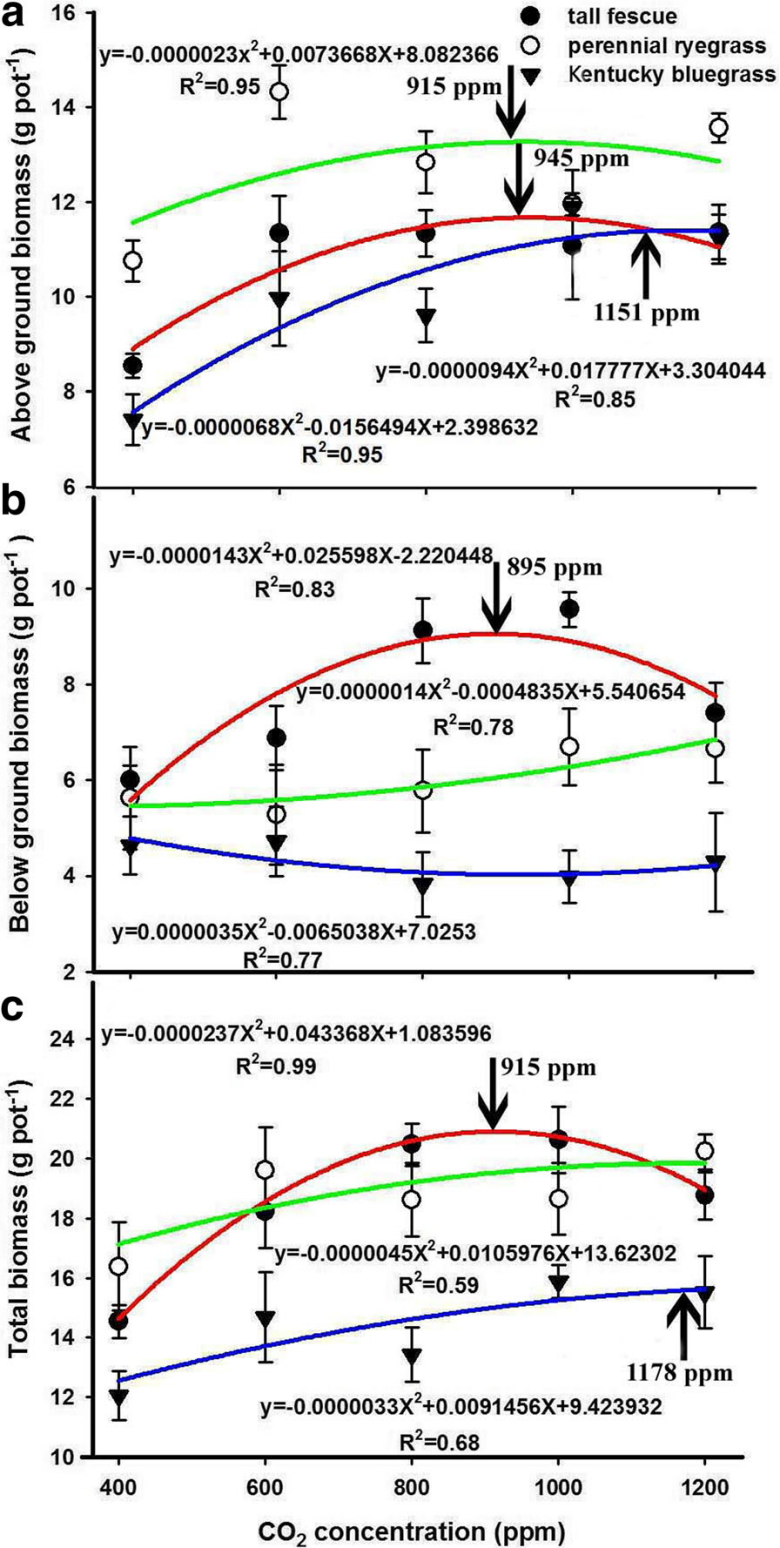
Effects of elevated CO_2_ concentrations on above ground biomass (**a**), below ground biomass (**b**), and total biomass (**c**) of the three grass species. Values given are mean ± standard deviation for *n* = 4 pots

We found very strong CO_2_ fertilization effects on the aboveground and total biomass of the three species. The optimal CO_2_ levels for the aboveground biomass were 945, 915, and 1151 ppm, and for the total biomass were 915, 1178, and 1386 ppm for tall fescue, perennial ryegrass, and Kentucky bluegrass, respectively (Fig. 1). However, an optimal CO_2_ of 895 ppm for the belowground was found only for the tall fescue, while no obviously optimal CO_2_ of the belowground biomass for the other two species was detected. Beyond the optimum, further elevating the ambient CO_2_ concentration significantly reduced the growth of perennial grasses, indicating the adverse impacts of high CO_2_ concentration on the grass species. Quadratic models can be used to adequately quantify the CO_2_ fertilization effect on the biomass of the three grasses (Fig. 1).

### Elevated CO_2_ effects on leaf gas exchange

**Fig. 2:**
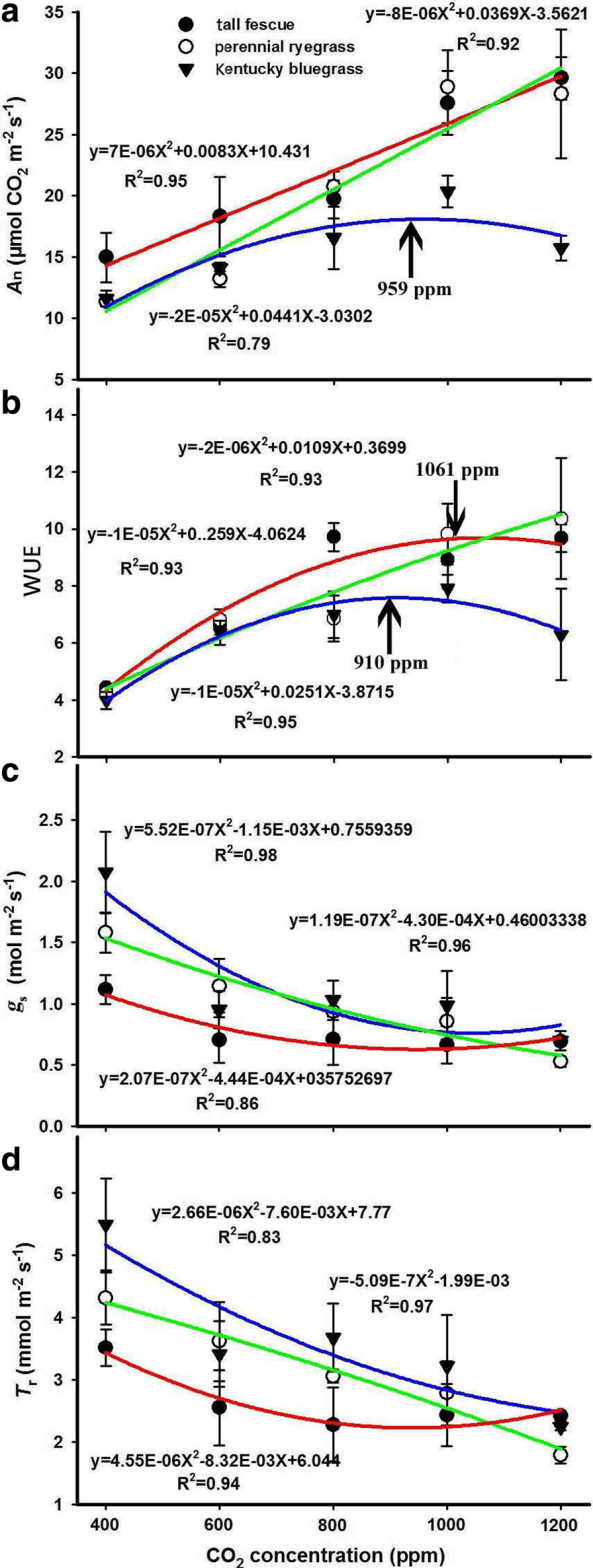
Effects of elevated CO_2_ concentrations on (**a**) leaf net photosynthesis rate (*A*_n_), (**b**) water use efficiency (WUE), (**c**) stomatal conductance (*g*_s_), and (**d**) transpiration rate (*T*_r_) of the three grass species. Values given are mean ± standard deviation for *n* = 4 pots

As with plant growth, the CO_2_ fertilization effect was also evident in the leaf net photosynthetic rate (*A*_n_) of Kentucky bluegrass, stimulating *A*_n_ by 75% when the CO_2_ increased from 400 ppm to 1000 ppm. The CO_2_ stimulation effect on *A*_n_ reached a maximum at 959 ppm, at which point further increase in CO_2_ resulted in a decline of *A*_n_ (Fig. 2). However, the response of *A*_n_ to elevated CO_2_ also varied with grass species. The leaf net photosynthetic rates of the other two species (tall fescue and perennial ryegrass) consistently increased with increasing CO_2_, which can also be described by quadratic relationships with optimal CO_2_ beyond the maximum CO_2_ treatment of this study. In contrast to *A*_n_, the stomatal conductance (*g*_s_) and transpiration rates (*T*_r_) of the three grasses decreased non-linearly with the increase of CO_2_ and the relationships of CO_2_-*g*_s_ and CO_2_-*T*_r_ also typically followed quadratic equations with maximum *g*_s_ and *T*_r_ occurring around 400 ppm, which was much lower than the optimal CO_2_ for plant growth and leaf photosynthesis.

As a result, the WUE of tall fescue and Kentucky bluegrass also featured bell-shaped curves in relation to CO_2_ concentration, with the maximum CO_2_ fertilization effect occurring at approximately 1062 ppm and 910 ppm, respectively. However, the maximum WUE of perennial ryegrass was beyond the highest CO_2_ concentration treatment of 1200 ppm. Thus, we quantified the relationship between CO_2_ and WUE of perennial ryegrass through quadratic models and found that the optimal CO_2_ for WUE would occur at about 2700 ppm, which was much higher than those of the other two species (Fig. 2).

**Fig. 3:**
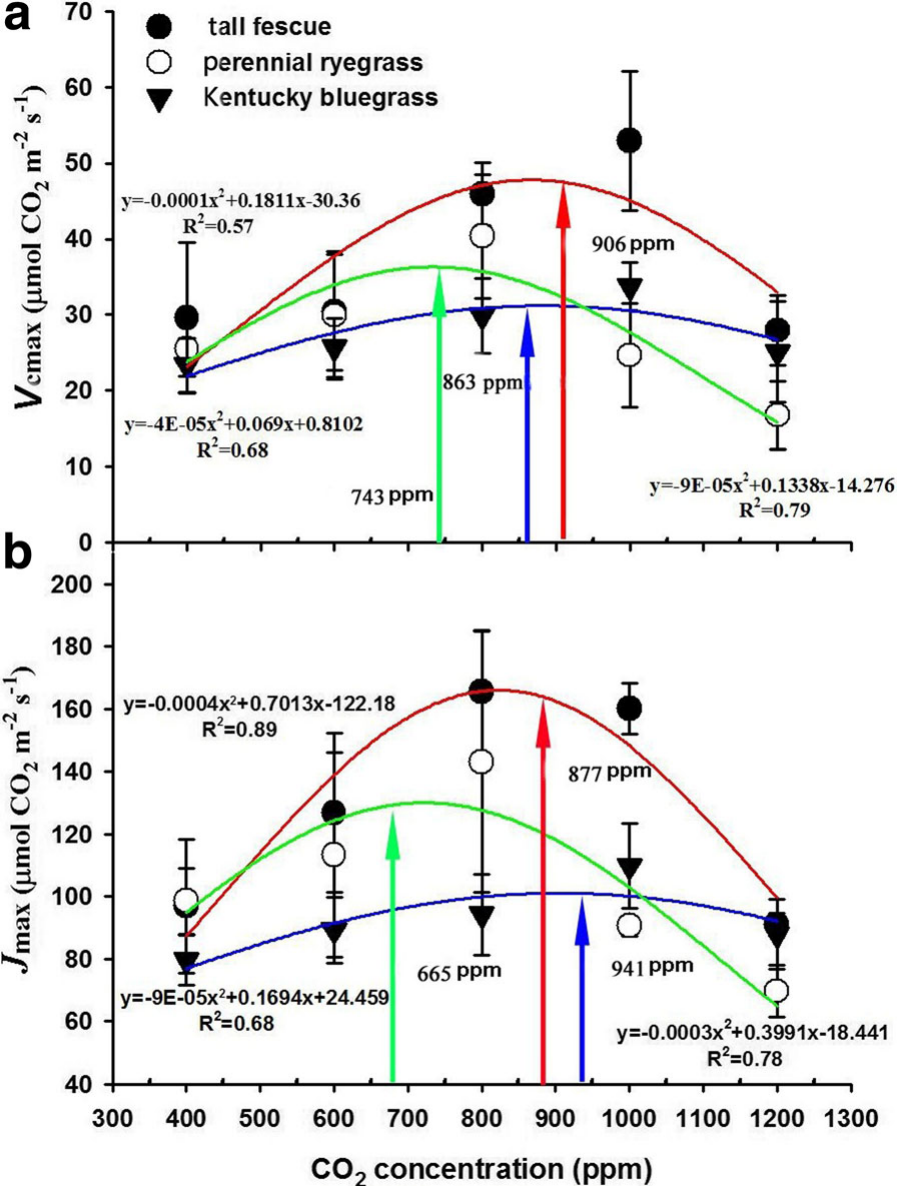
Effects of elevated CO_2_ concentrations on (**a**) the maximum carboxylation rate (*V*_cmax_) and (**b**) the maximum capacity of electron transport RuBP regeneration (*J*_max_) of the three grasses. Values given are mean ± standard deviation for *n* = 4 pots

The maximum carboxylation rate (*V*_cmax_) of the three grasses demonstrated bell-shaped curves in relation to CO_2_ concentration, peaking at 906 ppm, 863 ppm, and 743 ppm for tall fescue, perennial ryegrass, and Kentucky bluegrass, respectively (Fig. 3a). Similar to the *V*_cmax_, the maximum electron transport rate (*J*_max_) in response to increasing CO_2_ concentrations also shared bell-shaped curves for all three grasses. The optimal CO_2_ concentration of *J*_max_ was 877 ppm, 941 ppm, and 665 ppm for tall fescue, perennial ryegrass, and Kentucky bluegrass, respectively (Fig. 3b).

### Elevated CO_2_ effects on leaf dark respiration and non-structural carbohydrates

**Fig. 4:**
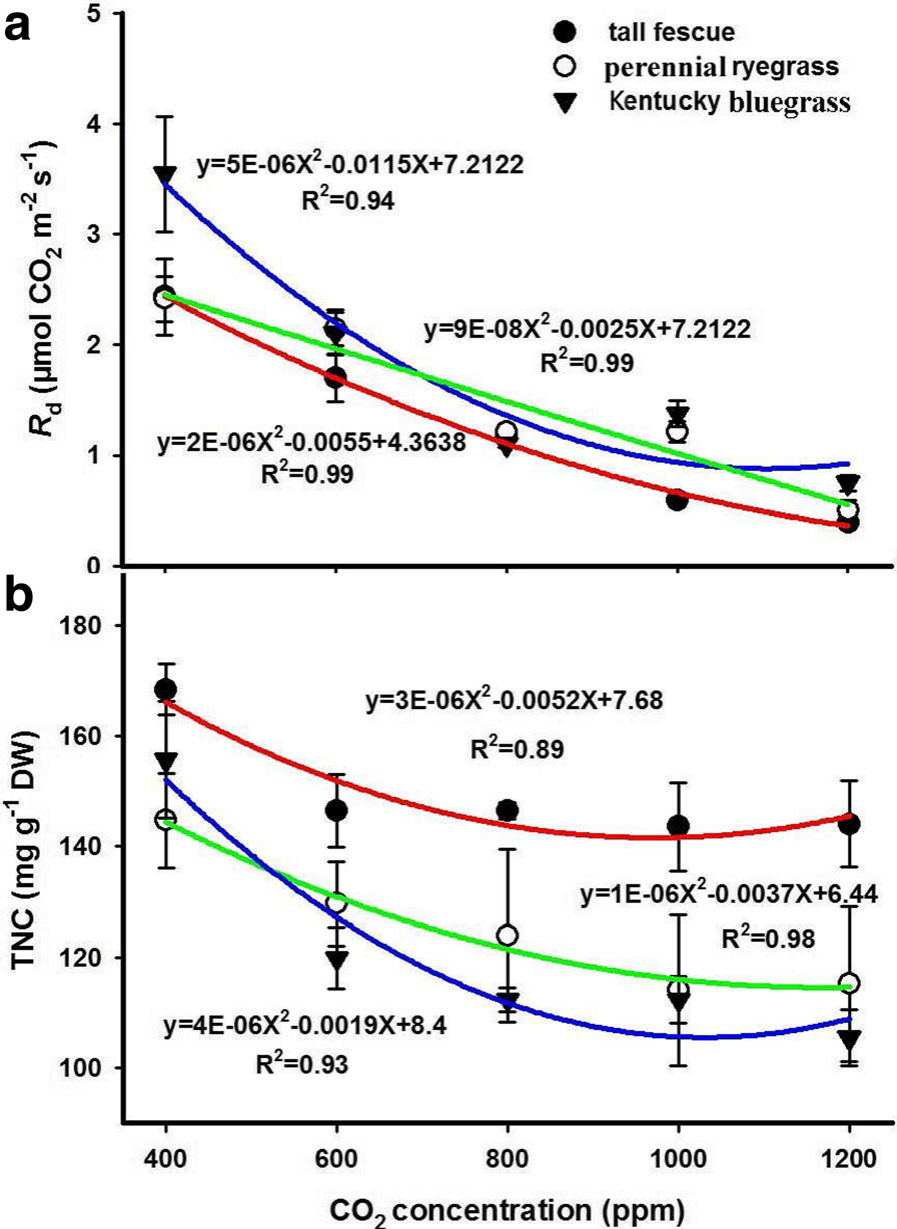
Effects of elevated CO_2_ concentrations on (**a**) the leaf dark respiration rates (*R*_d_) and (**b**) total non-structural carbohydrates (TNC) of the three grass species

**Fig. 5:**
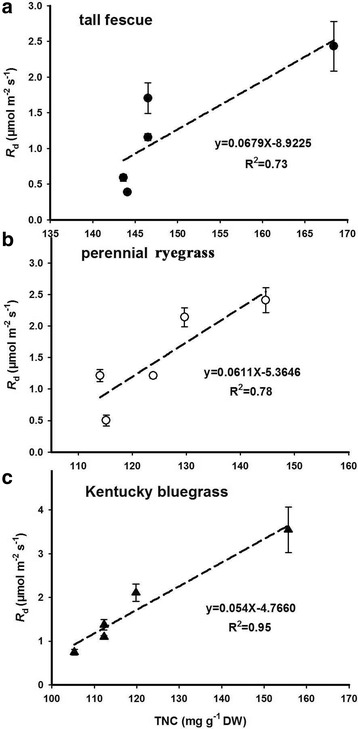
Effects of elevated CO_2_ concentrations on the linear relationship between TNC and *R*_d_ for tall fescue (**a**), perennial ryegrass (**b**), and Kentucky bluegrass (**c**)

Our results showed that leaf dark respiration (*R*_d_) of the three species substantially declined with increasing CO_2_ (Fig. 4). The relationships between *R*_d_ and CO_2_ of the three species were quantified through quadratic models with R^2^ values of 0.99, 0.99 and 0.94 for tall fescue, perennial ryegrass and Kentucky bluegrass respectively (Fig. 4a). Similar to the *R*_d_, the leaf total non-structural carbohydrate (TNC) of the three grasses also quadratically decreased with elevated CO_2_ (Fig. 4b). Meanwhile, we estimated the relationships between *R*_d_ and TNC (Fig. 5) and found that *R*_d_ was increased linearly by the enhancement of TNC, with R^2^ values such as 0.73, 0.78 and 0.95 for the tall fescue, perennial ryegrass, and Kentucky bluegrass, respectively.

### Elevated CO_2_ effects on tissue carbon (C) and nitrogen (N) contents and the relationships between leaf N and *V*_cmax_ or leaf N and *J*_max_

**Fig. 6:**
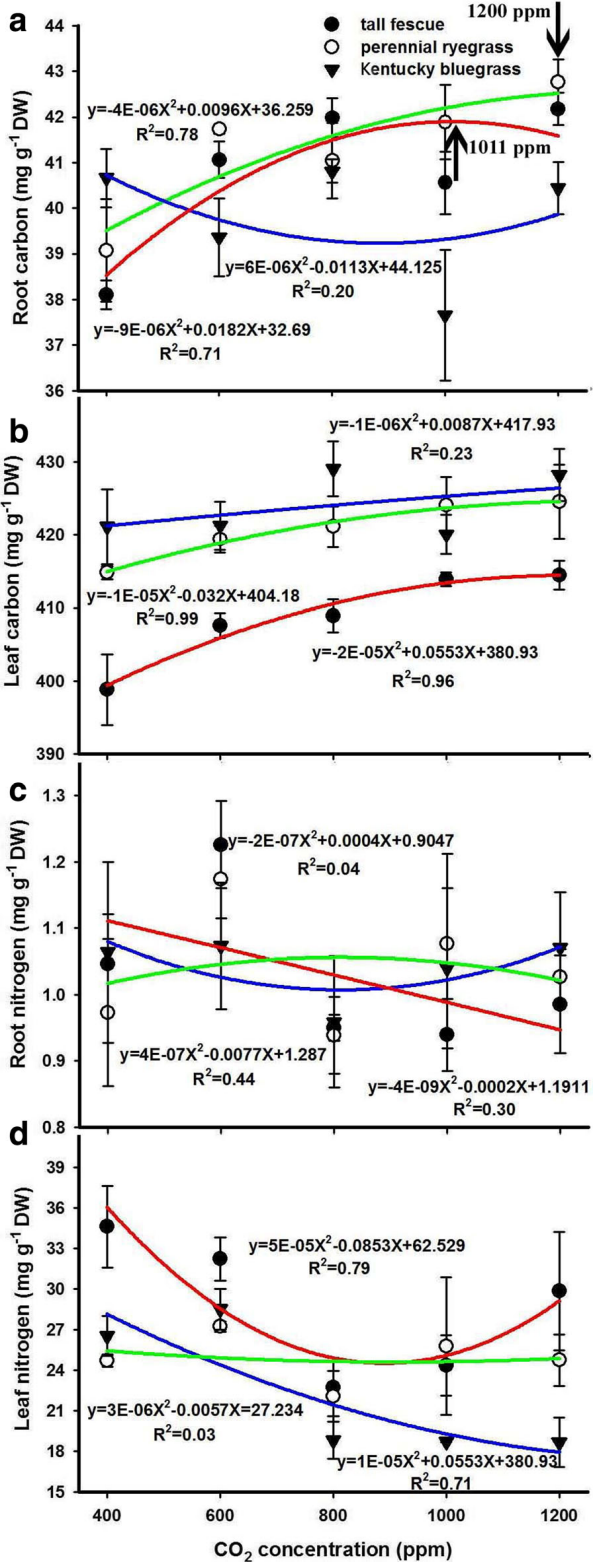
Effects of elevated CO_2_ concentrations on the carbon content of root (**a**) and leaf (**b**) as well as the nitrogen content of root (**c**) and leaf (**d**) of the three grass species. Values given are mean ± standard deviation for *n* = 4 pots

We found optimal CO_2_ concentrations in both the leaf and root of tall fescue and perennial ryegrass. The relationships between leaf carbon and CO_2_ featured bell-shaped curves with maximum values occurring at approximately 1388 and 1600 ppm for tall fescue and perennial ryegrass with R^2^ values 0.96 and 0.99 respectively (Fig. 6a). Interestingly, root carbon in response to elevated CO_2_ was also characterized by similar curves with R^2^ values 0.71 and 0.78 and optimal CO_2_ levels of 1011 and 1200 ppm for tall fescue and perennial ryegrass, respectively. However, we obtained very weak relationships between CO_2_ and tissue carbon with R^2^ values 0.23 for leaf and 0.19 for root of Kentucky bluegrass (Fig. 6a). In contrast to tissue carbon, both the leaf and root nitrogen of the tall fescue and Kentucky bluegrass quadratically decreased with elevated CO_2_ (Fig. 6b). By using the quadratic functions, we analyzed the relationships of leaf and root nitrogen with CO_2_ and found the R^2^ values to be 0.79 and 0.71, and 0.31 and 0.44 for the tall fescue and Kentucky bluegrass respectively (Fig. 6d). Our results also showed that elevated CO_2_ barely affected the tissue nitrogen of perennial ryegrass, evidenced by the weak quadratic relationships between CO_2_ and nitrogen with R^2^ values 0.03 and 0.04 for leaf and root respectively (Fig. 6c-d).

**Fig. 7:**
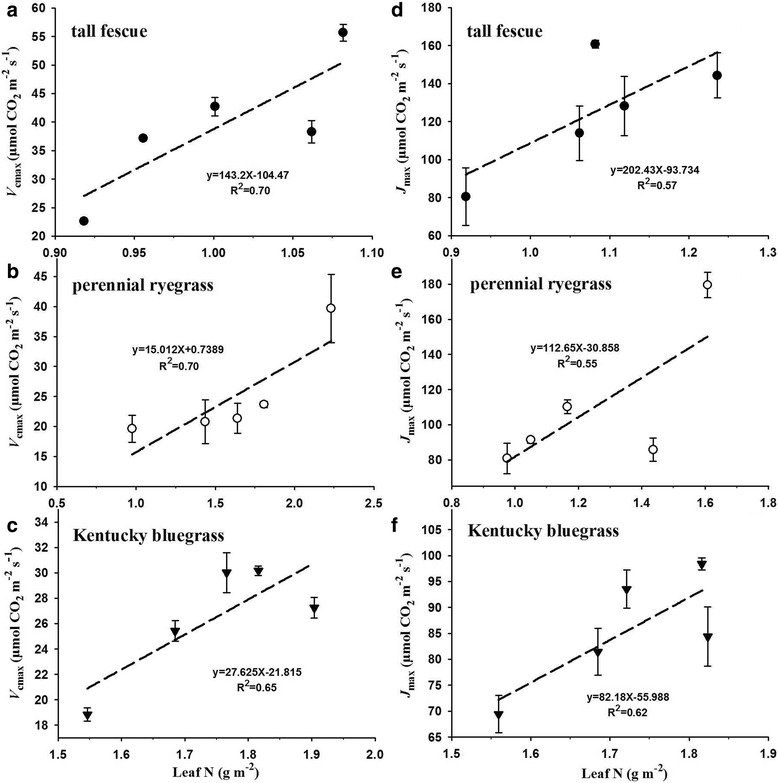
Effects of elevated CO_2_ concentrations on the relationships between *V*_cmax_ and leaf N (**a**-**c**) or *J*_max_ and leaf N (**d**-**f**) for the three grass species. Values given are mean ± standard deviation for *n* = 4 pots

We also evaluated the relationships between leaf N and *V*_cmax_ as well as leaf N and *J*_max_ of the three grass species (Fig. 7). Our results showed that the *V*_cmax_ values were linearly enhanced with the increases of leaf N for tall fescue (R^2^ = 0.70), perennial ryegrass (R^2^ = 0.70), and Kentucky bluegrass (R^2^ = 0.65, Fig. 7a-c). Similarly, we also found linearly positive relationships between leaf N and *J*_max_ with R^2^ values of 0.57, 0.55, and 0.62 for tall fescue, perennial ryegrass, and Kentucky bluegrass, respectively (Fig. 7d-f).

## Discussion

### Different optimal CO_2_ fertilization concentrations for the growth of perennial grasses

Most plants generally benefit from elevated atmospheric CO_2_ concentration through the “CO_2_ fertilization effect”, which boosts growth and yield [19, 23, 46, 52]. However, this positive CO_2_ fertilization effect strongly depends on the plant functional groups and species [7, 22, 53–56]. Even within the same species of winter wheat, the results from previous studies are inconsistent [22, 50, 57–60]. These contradictory results suggest that different plants and/or species may have different optimal CO_2_ concentrations for their growth. Our results showed that the optimal CO_2_ concentrations occurred at 945, 915, and 1151 ppm for the aboveground biomass and at 915, 1178, and 1386 ppm for the total biomass of tall fescue, perennial ryegrass, and Kentucky bluegrass (Fig. 1), suggesting that a strong CO_2_ fertilization effect occurred at different optimal CO_2_ concentrations for these three perennial grasses. This result also indicated that Kentucky bluegrass has the highest optimal CO_2_ concentration among the three grasses, and thus may suffer less from future climate change than the other two grasses. In addition, by enhancing the atmospheric CO_2_ concentration from 400 ppm to the optimum for each grass species, the maximum CO_2_ fertilization effect substantially increased the total biomass of the by 60%, 15%, and 30% for tall fescue, perennial ryegrass, and Kentucky bluegrass respectively. Interestingly, biomass enhancements of 15% and 30% for perennial ryegrass and Kentucky bluegrass are very similar with the average of approximately 20% for C_3_ plants as estimated in meta-analysis of Free-Air CO_2_ Enrichment (FACE) studies [61, 62], and 32% of Open Top Chamber (OTC) and greenhouse experiments [63]. However, the increased rate of tall fescue (60%) is much higher than those of the other two species, indicating this specie will benefit the most from the positive fertilization effect among these three perennial grasses under future high CO_2_ environmental conditions. It is noted that we found no obviously optimal CO_2_ for the belowground biomass of two species (Kentucky bluegrass and perennial ryegrass), as evidenced by the upward quadratic relationships between belowground biomass and CO_2_ concentrations. These results suggest that the carbon allocation between aboveground and belowground of the three grasses characterize different strategies, and tall fescue might select a more effective strategy to balance the carbon investment between aboveground and belowground than the other two species under high CO_2_ concentrations.

### The positive CO_2_ fertilization effect on the growth of perennial grasses

Previous studies have well demonstrated that plant growth is highly correlated with biochemical and photochemical processes [64, 65] such as photosynthesis and respiration, through which the CO_2_ fertilization effect is developed and regulated [22]. In the current study, the photosynthesis-CO_2_ relationship followed a similar bell-shaped curve like the biomass-CO_2_ relationship (Figs. 1 and 2), suggesting that the positive CO_2_ fertilization effect might be attributed to the up-regulation of *A*_n_, as evidenced by the increased leaf net photosynthetic rates (*A*_n_), with the maximum CO_2_ fertilization effect occurring at 959 ppm for Kentucky bluegrass, and 1200 ppm for both tall fescue and perennial ryegrass (Fig. 2a). Further analysis showed that leaf biochemical and photochemical processes played a key role in determining the positive CO_2_ fertilization effect through directly increasing both carboxylation rates and electron transport rates of perennial grasses. Our results showed that both the maximum carboxylation rate of Rubisco (*V*_cmax_) and the maximum capacity of electron transport RuBP regeneration (*J*_max_) of the three grasses were dramatically stimulated by elevated CO_2_ concentrations before reaching their optimums (Fig. 3), suggesting that the initial increase in CO_2_ concentration may favor both the light and dark reactions of photosynthesis through boosting the Rubisco carboxylation and the RuBP regeneration processes. Also, a recent study has reported that the *V*_cmax_ of winter wheat was dramatically increased by elevating ambient CO_2_ concentrations from 400 ppm to about 800 ppm [23].

In addition to leaf photosynthesis, the positive CO_2_ fertilization effect on the growth of perennial grasses may also closely associate with the changes in leaf respiration and total non-structural carbohydrates (TNC) under high CO_2_ concentrations. Our results showed that the leaf dark respiratory rates (*R*_d_) and leaf TNC of the three grasses consistently decreased with elevated CO_2_ concentrations. Meanwhile, we found a linear relationship between leaf *R*_d_ and TNC, suggesting that *R*_d_ reduction may partially attribute to decrease in leaf TNC, which is the most important substrate for leaf respiration [14, 66]. Overall, the up-regulation of *A*_n_ and the decline of *R*_d_ may both play pivotal roles in explaining the positive CO_2_ fertilization effects on the growth of perennial grasses in the current study.

### The diminishing returns of CO_2_ fertilization effect on perennial grasses

Previous studies have found that beyond certain thresholds, high CO_2_ concentration cause diminishing returns of CO_2_ fertilization effect on plants [13, 22, 23]. Several studies found that the stimulation of *A*_n_ induced by elevated CO_2_ decreased or even diminished if exposed for a longer time period, because plants acclimate to elevated CO_2_ concentrations through a process known as down-regulation [19, 32]. We also found bell-shaped curves for biomass-CO_2_ relationships for the three grasses similar to the *A*_n_-CO_2_, indicating a reduction in biomass due to a decline in the photosynthetic rate at high CO_2_ concentrations. It is well demonstrated that the down-regulation of *A*_n_ is possibly attributed to the changes in carbohydrates [31], under high CO_2_ environments. In the current study, elevated CO_2_ concentrations beyond the optima of the three grasses consistently reduced leaf TNC, suggesting that the imbalance of carbohydrate concentration in the source and sink was not a limiting factor for the down-regulation of *A*_n_. In addition, it is important to noted that hexokinase is a key functional enzyme for mediating sugar sensing [67] and may also decrease Rubisco content through inhibiting the expression of photosynthetic genes [68]. Previous studies have well demonstrated that the Rubisco content and activity of higher plants were dramatically decreased under high CO_2_ concentrations [68, 69], because leaf N was prior to enzymes relating to the metabolic processes of starch and sucrose than invested in Rubisco when plants was subjected to high CO_2_ concentrations [70]. Consequently, the changes in hexokinase with CO_2_ concentrations may contribute to the bell-shaped relationship between *A*_n_ and CO_2_ concentration, especially for the down-regulation of *A*_n_ under high CO_2_ concentrations.

It is well documented that stomatal conductance (*g*_s_) declines when exposed to elevated atmospheric CO_2_ concentration, and a doubling of CO_2_ from the present ambient concentration generally results in a reduction in *g*_s_ of 10–70% depending on species or functional groups [58]. In the current study, we also found that the *g*_s_ of all three grasses were dramatically decreased with elevated CO_2_ concentrations, which may be partly due to the down-regulation of *A*_n_ caused by CO_2_. Moreover, the reduced *g*_s_ under high CO_2_ concentrations might result in a decline in leaf transpiration and thus reduced nutrient availability, as observed in many previous studies [22]. Previous studies have claimed that elevated CO_2_ concentration increased plant C/N ratios mainly due to a decrease in N content [12, 26]. Similarly, we also found that the nitrogen contents of both tall fescue and Kentucky bluegrass were markedly decreased with increasing CO_2_ concentrations, which may also be caused by the CO_2_ effects on *A*_n_, since nitrogen content is associated with photosynthetic enzymes such as Rubisco [35–37]. In addition, the linearly positive relationships between leaf N and *V*_cmax_ for the three grasses (Fig. 7) were directly supporting the above conclusion that the down-regulation of *A*_n_ was partly attributed to the decline of leaf N under high CO_2_ concentrations.

It should be noted that the CO_2_ fertilization effect on plant growth may be confounded by future climate change such as global warming, nitrogen deposition, and drought, which may reduce or cancel out the CO_2_ fertilization effect [39]. For example, the global surface temperature may continue to increase and cause global precipitation to become unevenly distributed both temporally and spatially [2]. As a result, drought stress caused by the increased global surface temperature and the declined precipitation may also be a critical factor affecting leaf photosynthesis and respiration [17] and thus plant growth and biomass accumulation [49], and in turn the structure and function of ecosystems such as grasslands and pastures [37, 40]. Therefore, the fates of the three grasses cannot only be determined by elevated CO_2_ concentrations because warming and drought may have interactive effects with CO_2_ enhancement on the growth, physiological, and biological processes of the three grasses under future climate change [20]. Therefore, more controlled experiments with multiple factors such as temperature, drought, nutrition availability and CO_2_ concentration are needed for predicting the fates of grass species and thus the community dynamics of grasslands under future global climate change [31]. However, it is important to note that this study was carried out under controlled conditions with sufficient nutrients and water for plants during the experiment, which is obviously different from actual field conditions. Therefore, many similar experiments should be carried out in natural conditions without fertilization and watering for predicting the fates of the three cool-season C_3_ grasses in future climate change scenarios.

## Conclusions

We found that the optimal CO_2_ concentrations occurred at 945, 915, and 1151 ppm for the aboveground biomass of tall fescue, perennial ryegrass, and Kentucky bluegrass, respectively. Higher CO_2_ concentrations had diminishing returns of CO_2_ fertilization effect on plant growth, causing limiting effects on stomatal conductance, nitrogen availability and changes in the biochemical and photochemical processes of photosynthesis. Our results suggest that the continuously increasing atmospheric CO_2_ concentration in the future may dramatically lower the CO_2_ fertilization effect, and thus many current climate change models based on earlier results of “doubling–CO_2_” experiments may overestimate the CO_2_ fertilization effect on grasslands beyond the optimum CO_2_ concentration. According to recent IPCC reports, if global CO_2_ emissions are not effectively mitigated, the atmospheric CO_2_ concentration might be over 900 ppm in the second half of this Century. Nevertheless, the optimal CO_2_ concentrations found in this study can be used as an indicator in predicting the fates of the cool-season C_3_ grasses under future rising atmospheric CO_2_ concentration and climate change, because grasses with high optimal CO_2_ concentrations may take full advantage of the CO_2_ fertilization effect.

## Abbreviations

*A*_n_: net photosynthetic rates
*g*_s_: stomatal conductance
*R*_d_: dark respiration
TNC: total nonstructural carbohydrates
*T*_r_: transpiration rates
VPD: vapor pressure deficit
WUE: water use efficiency
